# Long-term treatment of somatostatin analog-refractory growth hormone-secreting pituitary tumors with pegvisomant alone or combined with long-acting somatostatin analogs: a retrospective analysis of clinical practice and outcomes

**DOI:** 10.1186/1756-9966-32-40

**Published:** 2013-06-21

**Authors:** Antonio Bianchi, Ferdinando Valentini, Raffaella Iuorio, Maurizio Poggi, Roberto Baldelli, Marina Passeri, Antonella Giampietro, Linda Tartaglione, Sabrina Chiloiro, Marialuisa Appetecchia, Patrizia Gargiulo, Andrea Fabbri, Vincenzo Toscano, Alfredo Pontecorvi, Laura De Marinis

**Affiliations:** 1 Department of Endocrinology, Catholic University, School of Medicine, Largo A. Gemelli 8, 00168, Rome, Italy; 2 Endocrinology Section, San Camillo-Forlanini Hospital, Via Portuense, 332A, 00151, Rome, Italy; 3 Endocrinology Section, University “La Sapienza”, Viale R. Elena, 324, 00185, Rome, Italy; 4 Endocrinology Section, St. Andrea Hospital, University “La Sapienza”, 2nd Faculty, Via di Grottarossa, 1035/1039, 00189, Rome, Italy; 5 Endocrinology Unit, Regina Elena Cancer Institute – IFO, Via Elio Chianesi 53, Rome, 00144, Rome, Italy; 6 Endocrinology Unit, St. Eugenio and CTO Andrea Alesini Hospital, University of Tor Vergata, Via San Nemesio, 21, 00145, Rome, Italy

## Abstract

**Background:**

Pegvisomant (PEGV) is widely used, alone or with somatostatin analogs (SSA), for GH-secreting pituitary tumors poorly controlled by SSAs alone. No information is available on specific indications for or relative efficacies of PEGV?+?SSA versus PEGV monotherapy. Aim of our study was to characterize real-life clinical use of PEGV vs. PEGV?+?SSA for SSA-resistant acromegaly (patient selection, long-term outcomes, adverse event rates, doses required to achieve control).

**Methods:**

A retrospective analysis of data collected in 2005–2010 in five hospital-based endocrinology centers in Rome was performed. Sixty-two adult acromegaly patients treated ≥6 months with PEGV (Group 1, n?=?35) or PEGV?+?SSA (Group 2, n?=?27) after unsuccessful maximal-dose SSA monotherapy (≥12 months) were enroled. Groups were compared in terms of clinical/biochemical characteristics at diagnosis and before PEGV or PEGV?+?SSA was started (baseline) and end-of-follow-up outcomes (IGF-I levels, adverse event rates, final PEGV doses).

**Results:**

Group 2 showed higher IGF-I and GH levels and sleep apnea rates, higher rates residual tumor tissue at baseline, more substantial responses to SSA monotherapy and worse outcomes (IGF-I normalization rates, final IGF-I levels). Tumor growth and hepatotoxicity events were rare in both groups. Final daily PEGV doses were similar and significantly increased with treatment duration in both groups.

**Conclusions:**

PEGV and PEGV?+?SSA are safe, effective solutions for managing SSA-refractory acromegaly. PEGV?+?SSA tends to be used for more aggressive disease associated with detectable tumor tissue. With both regimens, ongoing monitoring of responses is important since PEGV doses needed to maintain IGF-I control are likely to increase over time.

## Background

Elevated GH and IGF-I levels are major causes of morbidity and mortality in patients with acromegaly [1,2]. The mainstay of treatment involves surgical resection of the somatotrophic adenoma causing the disease. In experienced hands, it is associated with cure rates of 50-70%, depending on the size, morphology, and location of the tumor. Management of inoperable, residual, or relapsing disease is based on radiation and medical therapies. Long-acting somatostatin analogs (SSA), the drugs generally used for this purpose, restore “safe” levels of GH and IGF-I in 50-75% of acromegalic patients and produce some degree of tumor shrinkage in 50–80% [3-5].

Pegvisomant (PEGV), a pegylated recombinant human GH analog that acts as a GH-receptor antagonist, was approved by the European Medicines Agency in 2002 for treatment of acromegaly in patients with inadequate responses (or contraindications) to surgery and/or radiation therapy and to SSA monotherapy [6]. The indications approved in 2003 by the U.S. Food and Drug Administration were somewhat broader and included patients who could not be controlled (or tolerate) surgery and/or radiation and/or other medical therapies [7]. Numerous studies have documented PEGV’s efficacy in patients with persistent active acromegaly, with IGF-I normalization rates ranging from 63% to 97% [8-11].

Recent guidelines suggest that combination therapy with PEGV and an SSA (PEGV?+?SSA) may also be useful for patients whose acromegaly is poorly controlled by conventional approaches [5]. It has also been proposed as a more cost-effective alternative for patients who require high-dose PEG monotherapy [12-14]. A recent international survey [15] revealed that this approach is used in 94% of centers surveyed in the United States and 76% of those in Europe, and over 90% of the centers reported using combination therapy only after SSA monotherapy had failed. No information, however, is available on the criteria used by physicians in deciding to prescribe PEGV?+?SSA rather than PEGV monotherapy.

A small, short-term study by Trainer et al. found that the two approaches were equally effective in normalizing IGF-I levels in patients who are not controlled on SSA monotherapy [16]. Other investigators have suggested that PEGV?+?SSA might be useful to control tumor growth and improve glucose tolerance [13,14,17], but these hypotheses were not confirmed in subsequent studies [18-20]. Thus far, there have been no long-term prospective or retrospective studies directly comparing the outcomes of the two treatment regimens.

The aims of the present study were to characterize the use in five Italian hospitals of PEGV vs. PEGV?+?SSA regimens for the treatment of SSA-resistant acromegaly in terms of patient selection, long-term outcomes, adverse event rates, and doses required to achieve control.

## Methods

### Subjects, treatment, and follow-up protocols

We conducted a retrospective analysis of data collected between 1 March 2005 and 31 December 2010 in five hospital-based endocrinology centers in Rome, Italy. The protocol was approved by the Research Ethics Committees of each center, and all patients provided written, informed consent to review of their charts and publication of the study findings. Data were recorded on electronic forms by physicians involved in the patients’ care and sent to the Coordinating Center for analysis.

The inclusion criteria were: [1] active acromegaly [i.e. GH concentrations above 1 ng/ml after OGTT together with fasting plasma IGF-I concentrations above the normal ranges for age and sex; [2] treatment with long-acting SSA for at least 12 months at maximum tolerated dose [Octreotide LAR 30 mg/4 weeks or Lanreotide Autogel (ATG) 120 mg/4 weeks]; [3] resistance to SSA, defined by high serum IGF-I concentrations despite maximal dose of SSAs for at least 1 years, according to Colao and coworkers [21]; [4] treatment with PEGV alone or in addition to SSAs for at least 6 months; [5] available informations, before PEGV start, about the following evaluated and recorded comorbidities: hypopituitarism, hypertension, diabetes, cardiomyopathy, sleep apnea, vertebral fracture, goiter and colon cancer.

Pegvisomant (Somavert, Pfizer Italia S.r.l., Rome, Italy) mono- and combination-therapy regimens were prescribed by the attending physicians. The drug was administered subcutaneously, once or twice daily (depending on dose); loading doses were not used and starting dose was 10 mg/day s.c. in all patients. Dosage adjustments (± 5 mg/day ) were based on IGF-I responses after one month and every two months for the first year of treatment.

After the first year, patients were re-evaluated at least every six months and each visit included assays of serum IGF-I levels and serum transaminase levels (ALT and AST); pituitary imaging studies (magnetic resonance imaging [MRI]) were performed every year.

During the 6-year study period, all participating centers used the same assays (Immulite 2000, DPC, Los Angeles, CA) to measure GH (before PEGV start) and IGF-I concentrations (Interassay coefficients of variation: 5.5%–6.2% for GH assays, 6.4%–11.5% for IGF-1: detection limits: 0.01 μg/L and 0.2 μg/L, respectively). GH levels are measured in μg/L of IS 98/574 (1 mg corresponding to three international units somatropin) and are specified to be means of day curves (4 sampling time points collected over 2 hours).

### Data analysis and statistical methods

Enrolled patients were retrospectively divided into two groups: those who received PEGV monotherapy (Group 1) and those treated with PEGV?+?SSA (Group 2). To explore the rationale underlying physicians’ decision to prescribe the combination regimen, we compared the group characteristics at the time of diagnosis and at baseline (i.e., at the end of unsuccessful SSA monotherapy, right before PEGV therapy was started) (Table 1). IGF-I levels were analyzed as absolute concentrations and standard deviation scores (SDS) relative to normal age-adjusted adult values (normal range from −2 to?+?2 SDS). The formula used for the latter was: SDS?=?(In-value – mean of normal age-adjusted values)/standard deviation of mean of normal age-adjusted values) [22]. Baseline values had been measured with Immulite assays, but various assays had been used to measure values at the time of diagnosis. Therefore, changes in serum IGF-I levels from diagnosis to baseline (IGF-I ∆) were calculated using the IGF-I SDSs recorded at the two time points. Outcomes of PEGV therapy were assessed in terms of absolute IGF-I levels and SDS values recorded during follow-up. Safety was evaluated in terms of the percentage of patients who experienced significant CT- or MRI-documented adenoma enlargement (i.e., volume increase over baseline of?>?25% or at least 0.5 cc) [20]; significant elevations in serum ALT and/or AST (at least 1 test with values >3 times the upper limit of normal); and injection site events.

**Table 1:** Characteristics of groups 1 and 2 at acromegaly diagnosis and at baseline

		**All patients**	**Group 1 PEGV**	**Group 2 PEGV?+?SSA**
**A**	**Patients** – n (%)	62 ^a^	35 (56.4)	27 (43.6)
**T**	Males	21 (33.9)	11 (31)	10 (37)
				
**D**	Age at diagnosis (y) - median (range)	33 (18–72)	39 (21–72)	31 (18–70)
**I**	**Patients with macroadenomas** – n (%)	50 (83%)	28 (80)	22 (81.5)
**A**	**Comorbidities** - n (%)			
**G**	Hypertension	25 (40.3)	15 (42.8)	10 (37)
**N**	Diabetes	22 (35.5)	15 (42.8)	7 (25.9)
**O**	Cardiomyopathy	23 (37.1)	12 (34.2)	11 (40.7)
**S**	Sleep apnea	24 (38.7)	6 (17.1)	18 (66.6)*
**I**	Vertebral fractures	16 (25.8)	12 (34.2)	4 (14.8)
**S**	Goiter	23 (27.1)	12 (34.2)	11 (40.7)
	Colon cancer	3 (4.8)	1 (2.8)	2 (7.4)
	**Hypopituitarism** – n (%)	27 (43.5)	13 (37.1)	14 (51.8)
	ACTH deficiency	4 (6.5)	2 (5.7)	2 (7.4)
	LH/FSH deficiency	25 (40.3)	13 (37.1)	12 (44.4)
	TSH deficiency	7 (11.3)	5 (14.2)	2 (7.4)
	Vasopressin deficiency	0 (0)	0 (0)	0 (0)
	**Hyperprolactinemia** – n (%)	12 (19.3)	6 (17.1)	6 (22.2)
	**GH nadir -** μg/L ^b^			
	Median (range)	10.25 (2.2-100)	9.4 (2.2-63.1)	17.1 (3.3-100)*
	Mean (±SD)	22.2 (±23)	16.9 (±17.3)	29 (±27.6)*
	**IGF-I levels**			
	μg/L, Median (range)	715 (315–1587)	670 (315–1210)	899 (425–1587)*
	SDS (range)	9.9 (2.9-22.2)	8.8 (2.9-22.2)	10.9 (3.6-21.7)*
	ng/ml, Mean (±SD)	804 (±246)	723 (±216)	906 (±254)
**A**	**BMI** (kg/m^2^) – median (range)	28.7 (19.1-42)	27 (20–42)	30 (19.1-37.8)
**T**	**Estimated disease duration (y)** – median (range)	5 (2–20)	5 (2–20)	5 (2–20)
				
**B**	**Previous treatments** – n (%)			
**A**	Surgery – n (%)	59 (95.2)	33 (94.2)	26 (96.3)
**S**	Residual adenoma	39 (62.9)	17 (51.5)	22 (84.6)*
**E**	Somatostatin analogs - n (%)	62 (100)	35 (100)	27 (100)
**L**	Duration of treatment (y) – median (range)	4 (2–17)	4 (2–16)	4 (2–17)
**I**	Radiotherapy - n (%)	16 (25.8)	7 (20)	9 (33)
**N**	Dopamine agonists - n (%)	13 (20.9)	7 (20)	6 (22)
**E**^**c**^	**GH levels** - μg/L ^d^			
	Median (range)	11 (0.8-77)	8.4 (0.8-77)	18 (3.8-74.0)*
	Mean (±SD)	21.4 (±21)	17.2 (±19.7)	30.9 (±22.5)*
	**IGF-I levels**			
	μg/L , Median (range)	621.5 (431–1621)	632 (431–1621)	592 (455–929)^#^
	SDS (range)	6.9 (2.7-19.5)	6.9 (2.7-19.1)	5.9 (3.4-16.5)^#^
	μg/L , Mean (±SD)	673(±224)	736 (±258)	661 (±162)^#^
	Δ** IGF-I**^e^			
	μg/L , Median (range)	132 (−411-872)	57 (−411-692)	205 (−115-872)*
	SDS (range)	2 (−5.8-13.4)	0.9 (−5.8-11.2)	3.1 (−1.7-13.4)*
	μg/L , Mean (±SD)	131 (±266)	38 (±250)	251 (±241)*

The results are shown as median (range) or number (percent), unless otherwise specified. Abbreviations: *BMI* body mass index, *PEGV* pegvisomant, *SSA* somatostatin analogs, *SDS* standard deviation score.

Systeme Internationale conversion factors: GH (μg/L), X 3.0?=?mUI/L; IGF-I (μg/L), X 0.131?=?nmol/L.

^a^ Nineteen were analyzed in the Acrostudy Italy;

^b^ GH nadir?=?value observed after oral glucose tolerance test (OGTT);

^c^ Baseline: End of SSA monotherapy, immediately before PEGV was started.

^d^ Expressed as averages of GH day curve (4 points over 2 hours).

^e^ Level observed at diagnosis minus level observed at baseline.

* p?<?0.05 vs. Group 1 (Mann–Whitney U test); # p?<?0.05 vs. diagnosis (Wilcoxon rank sum test).

Intragroup differences involving continuous variables were analyzed with the Wilcoxon rank sum test; the Mann–Whitney U test when data from different groups were being compared. For discontinuous variables, the chi-squared test was used. Multivariate logistic regression analysis was used to identify factors related to the decision to prescribe PEGV?+?SSA vs. PEGV monotherapy. Standard and stepwise multiple linear regression analyses were used to identify variables that best predicted the end-of-follow-up PEGV dose. P values <0.05 were regarded as significant.

## Results

The study population included 62 patients with acromegaly caused by GH-secreting adenomas (Table 1). The vast majority had presented with macroadenomas. Almost all had already undergone surgery, but at baseline 2/3 had detectable residual adenoma. Three patients were treated with SSA as primary therapy: in two cases because the neurosurgery was contraindicated due to severe cardiomyopathy and respiratory comorbidities and in the last case the patient refused surgery. All had received?≥?2 years of SSA monotherapy. All patients were on SSA treatment [octreotide LAR n?=?23 (37%), lanreotide ATG n?=?39 (63%)] before PEGV replaced or was added to SSA.

Laboratory data obtained right before this treatment was discontinued (i.e., baseline) revealed the persistence of markedly elevated GH (median nadir 18 μg/L) and IGF-I levels (median 621 μg/L). The mean IGF-I ∆ was 132 μg/L (range −411 to 872).

Thirty-five of the patients had been treated with PEGV alone (Group 1) and 27 were receiving PEGV?+?SSA (Group 2), continuing the previous SSA treatment. As shown in Table 1, median GH and IGF-I levels documented at the time of diagnosis were significantly higher in Group 2 (p?<?0.05 vs. Group 1), but the frequencies of hypopituitarism in the two groups were similar. Rates of individual comorbidities were also similar, with the exception of sleep apnea, which was more common in Group 2. At baseline, there were no significant intergroup differences in disease duration, BMIs, or treatment histories. Before PEGV, no differences in octreotide LAR and lanreotide ATG treated patients were found between the two groups [Group 1: octreotide LAR?=?14 (40%), Lanreotide ATG?=?21 (69%) patients; Group 2: octreotide LAR?=?9 (33%), Lanreotide ATG?=?18 (67%)]. However, Group 2 had significantly higher residual tumor rates and (as at diagnosis) GH levels that were nnearly twice as high as those of Group 1. Baseline IGF-I levels in both groups still clearly exceeded normal ranges. However, the IGF-I ∆ values (SDS) in Group 2 were 3–4 times higher than that of Group 1. As a result, when SSA monotherapy was discontinued (i.e., baseline), the IGF-I elevations in the two groups were not significantly different (Table 1). Multivariate logistic regression analyses revealed that the decision to prescribe PEGV?+?SSA vs. PEGV was significantly correlated with the presence of detectable tumor at baseline (p?=?0.002) and with the IGF-I response to previous therapy reflected in the ∆ IGF-I (p?=?0.001) (Table 2).

**Table 2:** Logistic regression analysis: variables determining the decision to prescribe PEGV with or without SSA therapy (dependent variable)

**COVARIATES**	**OR (95% CI)**	**P**
**GH at baseline** (μg/L)	1.015 (0.983-1.043)	1.047
**IGF-I SDS at baseline**	1.003 (0.999-1.007)	0.097
Δ** IGF I**^**a**^**SDS**	1.446 (1.153-1.814)	0.001
**Detectable adenoma at baseline**^b^	13.757 (2.547-74.307)	0.002

Abbreviations: *CI* confidence intervals, *OR* odds ratios, *PEGV* pegvisomant, *SSA* somatostatin analogs.

^a^ SDS observed at diagnosis minus SDS observed at baseline.

^b^ Includes patients who had not had surgery and those who had undergone surgery but presented residual tumor at baseline.

 Table 3 shows the treatment outcomes and adverse effects (AEs) reported during follow-up. The duration of PEGV therapy was significantly longer in Group 1 (p?<?0.05), but the daily doses being administered in the two groups at the end of follow-up were similar. Both regimens were generally well tolerated (Table 3). None of the patients on monotherapy displayed significant tumor growth, and in one case MRI documented progressive shrinkage of the adenoma, which was no longer detectable after 6 years of treatment. In Group 2, significant growth (> 25%) of residual adenoma tissue was observed in only one case. The patient had always had very aggressive disease that was difficult/impossible to control, and when the tumor enlargement was noted, he was receiving PEGV 40 mg/day plus lanreotide ATG 120 mg every 4 weeks. Eight (12.9%) patients (five in Group 1, three in Group 2) experienced significant hypertransaminasemia. Six of these had diabetes, and five had elevated IGF-I levels at end of follow-up. Daily PEGV doses at the time of the hypertransaminasemia varied: three patients were receiving 30 mg, four were taking 15 mg, and one was on 10 mg /day. All episodes resolved spontaneously without treatment interruption or dose reductions. Two AEs at the injection site were observed (one in each group).

**Table 3:** End-of-follow-up findings in Groups 1 and 2

	**Group 1 PEGV**	**Group 2 PEGV?+?SSA**
**Patients** – n (%)	35 (56.4)	27 (43.6)
**Duration (mo.) of PEGV therapy** – median (range)	51 (15–72)	30 (6–72)*
**Final weekly PEGV dose (mg)** – median (range)	105 (70–210)	140 (70–280)
**Final daily PEGV dose (mg)**		
**10 mg** – n (%)	10 (28.6)	11(40.7)
**15 mg** – n (%)	11 (31.4)	2 (7.4)
**20 mg** – n (%)	9 (25.7)	8 (29.6)
**25 mg** – n (%)	1 (2.8)	1 (3.7)
**30 mg** – n (%)	4 (11.4)	4 (14.8)
**40 mg** – n (%)	0 (0)	1 (3.7)
**Group mean (±SD)**	16.8 (±6.3)	17.9 (±8.4)
**Group median (range)**	15 (10–30)	20 (10–40)
**Subgroup with IGF-I normalization at end of follow-up**	15 (10–30)	10 (10–30)
**Subgroup with abnormal IGF-I levels at end of follow-up**	15 (10–20)	20 (10–40)*^#^
**Pts. requiring dose reduction during follow-up**^a^**– n (%)**	5 (14.3)	4 (14.8)
**Pts. with IGF-I normalization at any time during follow-up**^b^**– n (%)**	29 (82.8)	18 (66.7)
**Pts. with IGF-I normalization at end of follow-up** – n (%)	28 (80)	15 (55.5)*
**Final IGF-I levels**		
μg/L,Median (range)	212 (110–1216)^#^	291 (150–1015)*^#^
SDS (range)	1.0 (−0.5–14.1)^#^	1.9 (−0.4–9.8)*^#^
μg/L,Mean (±SD)	269 (± 203)	372 (± 216)*^#^
**Significant growth of (residual) adenoma** - n (%)	0 (0)	1 (3.7)
**Increase of liver enzymes** - n (%)	5 (14.3)	3 (11.1)
**Injections site events** - n (%)	1 (2.9)	1 (3.7)

^a^ For these patients alone, final doses do not necessarily correspond to maximal doses.

^b^ Includes pts. whose IGF-I levels were not normalized at the end of follow-up.

* p?<?0.05 vs. Group 1; ^#^?=?p?<?0.05 vs. baseline IGF-I levels.

The results are shown as median (range) or number (percent), unless otherwise specified. Systeme Internationale conversion factors: IGF-I (μg/L) X 0.131?=?nmol/liter.

It is important to note that in most cases the final doses shown in Table 3 are also the maximum doses prescribed for the patients. In 9 cases (five in Group 1, four in Group 2), however, PEGV doses that initially normalized IGF-I levels had to be reduced later because values dropped below the normal range. In Group 1, the dose reduction was followed by IGF-I re-normalization in 4 cases and increases to abnormally high levels in the fifth. In contrast, re-normalization was observed in only 1 of the 4 patients in Group 2 whose doses had been decreased: in the other 3 cases, the dose reduction resulted in end-of-follow-up levels that exceeded normal limits. IGF-I normalization was thus achieved at least once during follow-up in 47 (75.8%) patients, but only 43 (69.4%) of these were still controlled at the end of follow-up. As shown in Table 3, the latter outcome was significantly more common in Group 1 (p?<?0.05).

End-of-follow-up IGF-I values (Table 3) were also significantly lower in Group 1, although both groups experienced significant reductions relative to baseline levels (see Table 1). As shown in Table 3, analysis of the PEGV doses in subgroups with normal and elevated IGF-I levels at the end of follow-up revealed no significant differences between the normalized subsets of Groups 1 and 2. However, in Group 2 patients whose end-of-follow-up IGF-I levels were still elevated, the final PEGV doses were significantly higher than those used in non-normalized patients in Group 1. Indeed, this subset was the only one in which the median dose increased significantly as compared to that prescribed at baseline.

**Figure 1:**
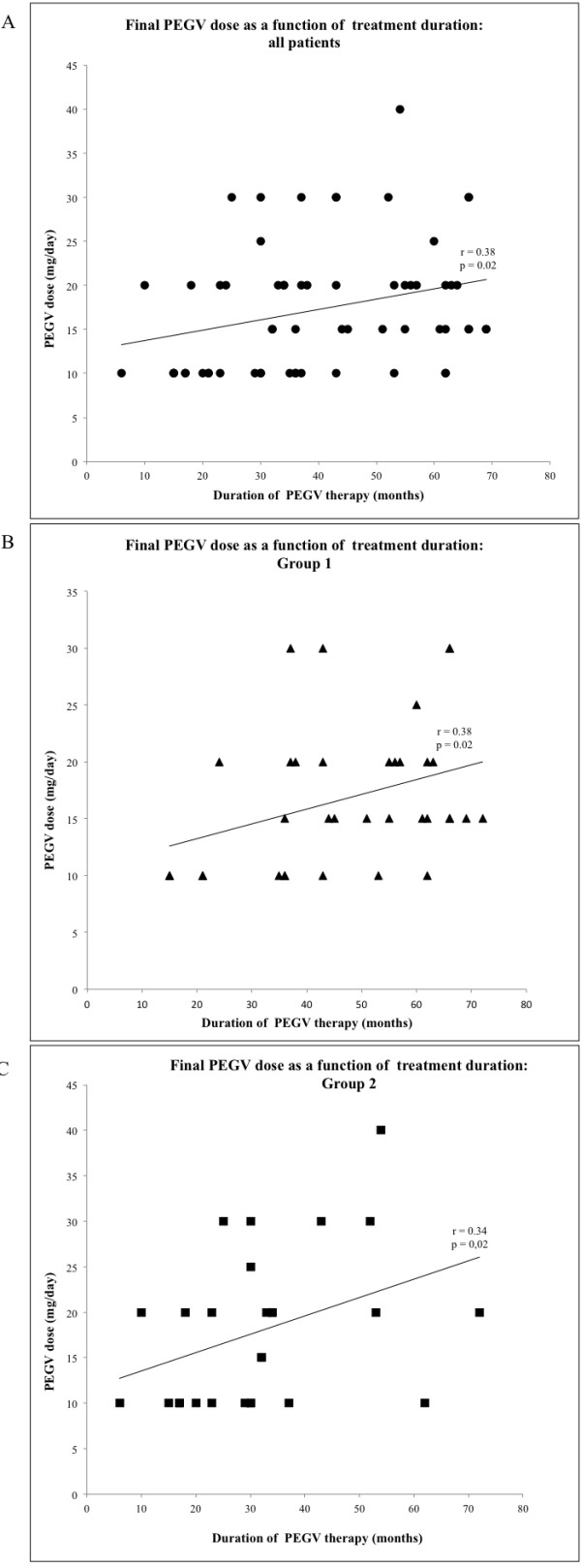
**Relationship between duration of PEGV therapy and final daily dose according to treatment regimen.** Correlation between duration of PEGV therapy (months) and final daily PEGV dose (mg/day) in the total study population (**A**, upper panel, ●), Group 1 (**B**, middle panel, ■), and Group 2 (**C**, lower panel▲). Regression coefficients (r) and p values are shown.

**Figure 2:**
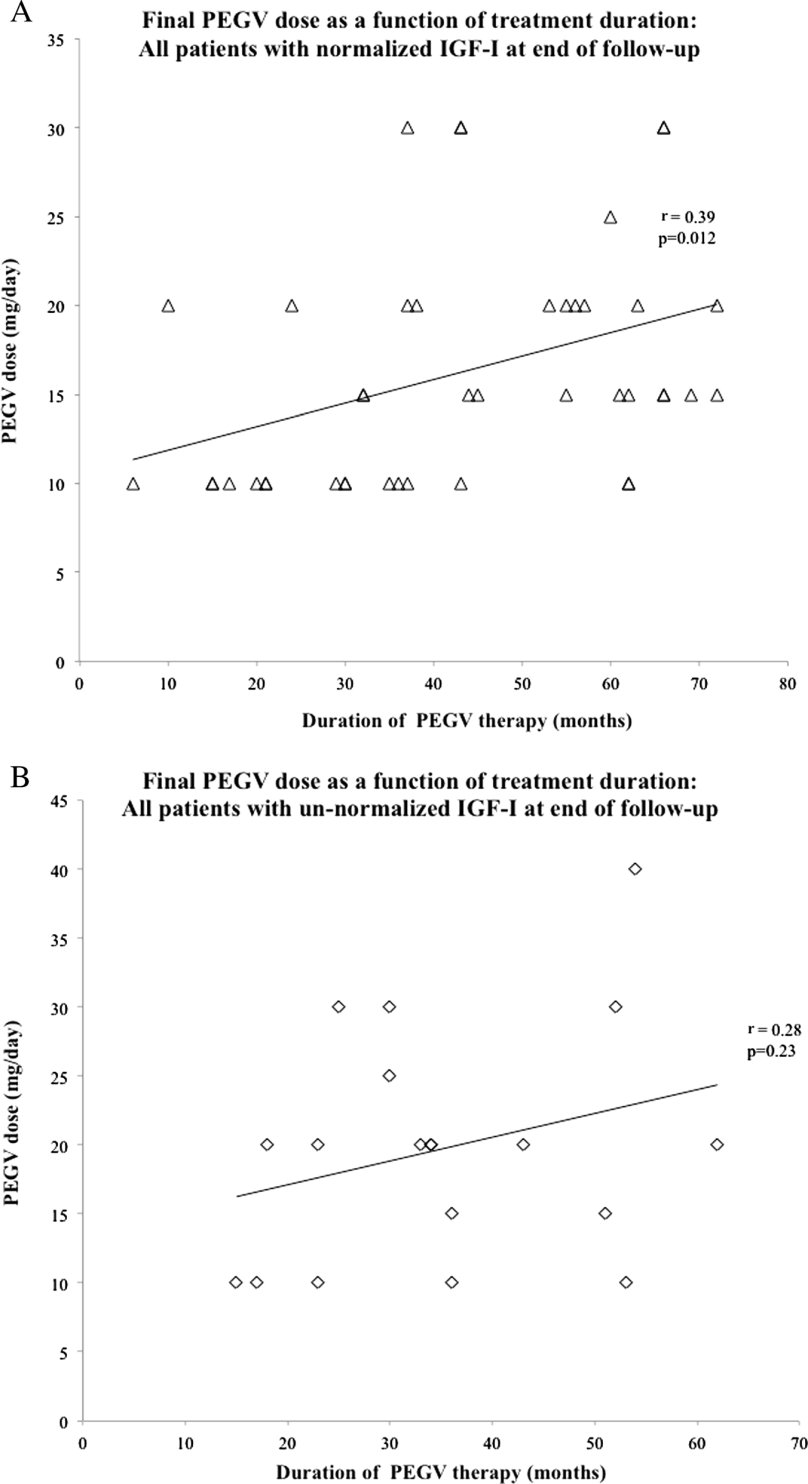
**Relationship between duration of PEGV therapy and final daily dose according to outcome.** Correlation between duration of PEGV therapy (months) and final daily PEGV dose (mg/day) in all patients (both groups) with IGF-I normalization at the end of follow-up (**A**, upper panel, ◊) and all patients (both groups) with non-normalized IGF-I levels at the end of follow-up (**B**, lower panel, Δ). Regression coefficient (r) and p value are shown.

To identify factors influencing the daily PEGV dose being used at the end of our follow-up, we performed multiple linear regression analysis using standard and stepwise methods. The covariates included in the model were treatment regimen (PEGV vs. PEGV?+?SSAs), detectable adenoma at baseline, baseline GH level, ∆ IGF-I SDS, sex, previous radiotherapy, and duration of PEGV therapy. Treatment duration was the only factor significantly correlated with the final PEGV dose, regardless of whether it was expressed in milligrams per day (standard regression: B?=?0.451±0.059; p?=?0.017; stepwise regression: B?=?0.117±0.052; p?=?0.026) (Figure 1) or in milligrams per day per BMI (standard regression: B?=?0.004±0.002; p?=?0.031; stepwise regression: B?=?0.004±0.022; p?=?0.025). Longer treatment was associated with significantly higher daily doses when Groups 1 and 2 were analyzed together (Figure 1A) or separately (Figure 1B-C). The correlation was also significant when we analyzed all patients from Groups 1 and 2 whose final IGF-I levels were normal (Figure 2A), but not when analysis was limited to patients whose final IGF-I levels exceeded normal ranges (Figure 2B).

## Discussion

This retrospective, observational study was conducted in 5 Italian hospitals to characterize the use of PEGV vs. PEGV?+?SSA regimens to manage SSA-resistant acromegaly. We found that combination therapy was more likely to be prescribed for patients with clinical/biochemical/imaging evidence of relatively severe/aggressive disease along with a more substantial (albeit incomplete) IGF-I response to SSA monotherapy. Both regimens were well tolerated, and at the end of follow-up, there was no significant difference between the daily PEGV doses in the two groups. However, outcomes (IGF-I normalization rates and final IGF-I SDS) were significantly worse in the patients receiving PEGV?+?SSA. The only variable significantly related to the final PEGV doses in both groups was treatment duration.

Given the size and nature of our sample, it is difficult to tell whether and to what extent our observations on prescribing practices are indicative of practices in other hospitals in Italy or other countries. The tendency to prescribe PEGV?+?SSA for acromegaly patients with more severe disease has not emerged from previous studies [8,9,12,13,16,23,24]. The only difference noted by Filopanti et al. in the Italian cohort they investigated was that patients on PEGV?+?SSA were more likely to have had macroadenomas at the time of diagnosis [24]. This was not observed in our population, although our Group 2 patients did have higher postoperative rates of residual tumor tissue. The increased disease severity in Group 2 was manifested by GH and IGF-I levels at diagnosis that were significantly higher than those in the group treated with PEGV alone. Our two treatment groups—like those analyzed by Reid et al. [25]—also had similar comorbidity rates when the disease was diagnosed. The exception was sleep apnea, which was roughly three times more common in Group 2. This disorder is being reported with increasing frequency in acromegaly patients [25], and its correlation with disease activity (IGF-I levels) has been demonstrated [26]. According to Roemmler et al. [26], our data confirm that sleep apnea is a frequent problem among patients whose disease is poorly controlled, especially those who present with more severe disease activity.

Clear-cut guidelines on the selection of patients for PEGV?+?SSA therapy (instead of PEGV alone) are lacking, although Melmed et al. note that combination therapy might be more cost-effective in patients who would otherwise require high-dose PEGV monotherapy [5]. In our population, the decision to use PEGV?+?SSA was significantly influenced by the extent of the IGF-I reduction observed after?≥?12 months of SSA monotherapy, which was approximately three times higher in Group 2 than in Group 1. This may reflect prescribers’ belief that, as suggested by Colao et al. [21], the efficacy of SSA therapy (in terms of biochemical control and limitation of tumor growth) may emerge only after several years of therapy, particularly when at least some positive effects have been observed with SSA monotherapy. The most important factor in prescribing decisions, however, was the presence or post-operative persistence of MRI-documented tumor tissue. Recent data indicate that the fear of increased tumor growth during PEGV monotherapy is unfounded [19,27], and our experience confirms this conclusion. Significant increases in tumor volume were extremely rare during follow-up (median duration 37 months) and showed no relation to the treatment regimen (PEGV vs. PEGV?+?SSA). Transaminase elevation rates were also low, which is consistent with previous reports [11,27], and, as noted by other investigators [17], these episodes occurred mainly in diabetics.

The IGF-I normalization rates observed in the two groups were in line with those recently reported by Van der Lely et al. [11]. They differ, however, from those reported in other studies, involving patients who had less severe disease at baseline than ours (especially those on combination therapy) and were followed for shorter periods of time. In these studies IGF-I normalization rates achieved with PEGV and PEGV?+?SSA often exceeded 90%, especially in the early studies with follow-ups of <52 weeks [8,9,12,13] but also in the long-term study conducted by Neggers et al. [14]. Rates more similar to our own were reported in 2011 by Van der Lely et al. [23] in patients with “partial” SSA-resistance treated PEGV?+?SSA: 78.9% achieved IGF-I normalization at least once, and 58% were still controlled at the end of follow-up. The final PEGV doses in that study were far lower than those recorded in our population, reflecting once again the severity of the disease in our patients. Inadequate dosing by the prescribing physician and/or poor patient compliance may also have contributed to the lower-than-expected normalization rates we observed. As noted by other authors [11], dose increases to?>?20 mg/day sometimes meet with poor compliance because they require two injections a day.

In contrast to recent data reported by Neggers et al. [28], we—like VanderLely et al. [11]—found no significant differences between the PEGV and PEGV?+?SSA treatment groups in terms of the PEGV doses used or the number of patients controlled. At the time of diagnosis, Group 2 patients had more marked biochemical derangements than those of Group 1, but when SSA monotherapy was discontinued, the GH and IGF-I levels of the two groups were similar. However, the same dose of PEGV appears to have been more effective when administered alone than it was when administered with an SSA. In all probability, this was due mainly to the fact that patients who received PEGV?+?SSA had more aggressive disease.

Treatment duration was significantly longer in patients being managed with PEGV monotherapy. Many of these were among the first in Italy to be treated with PEGV, and they may well have been selected precisely because their disease was relatively mild, with small tumors / residual tumors and IGF-I and GH levels considered more likely to be controlled safely by the new drug (based on data available at that time). It is important to recall that we did not analyze the reasons for the two groups’ different responses to SSA monotherapy. Multiple biochemical and clinical factors are known to influence the response to these drugs [21], and an analysis of this type was beyond the scope of our study. In contrast with the findings of Trainer et al. [29], the final PEGV doses being used by patients who were not controlled (in either group) were no lower than those of the patients with normal IGF-I levels at the end of follow-up. Within Group 2, PEGV doses for the uncontrolled subset of patients were higher than those being used by the normalized subset, which suggests that attempts had been made (albeit unsuccessfully) to achieve control by dose increases. Previous short-term [30,31] and long-term [32] studies have demonstrated that the PEGV dose required for IGF-I normalization is influenced by various factors, including body weight, sex, previous radiotherapy, baseline GH and IGF-I levels, and GH-receptor (GHR) polymorphisms, although a more recent study failed to confirm the importance of the last factor in responses to PEGV or to PEGV?+?SSA [24]. According to other authors [24], our data showed that both monotherapy or combination and final dose of PEGV are not affected by previous radiotherapy, probably because that was performed only in about 26% of patients, whereas the same treatment was reported in a high proportion of patients (58-66%) in previous studies [30,32]. Our findings are the first that reveal a strong linear relation between the IGF-I-normalizing dose and the duration of PEGV treatment, regardless of whether the latter is combined with SSAs. This correlation was not significant in patients who failed to achieve IGF-I normalization at the end of follow-up, probably because these patients had more aggressive disease with higher levels of GH. Inadequate dose adjustment may also have played a role.

Previous studies [8,9,11] indicate that the percentage of patients controlled by PEGV remains stable over time. The earliest studies, which were short-term trials, showed that higher doses were associated with proportionally higher control rates, and that the dose required to achieve normalization depended on pre-PEGV IGF-I levels [14,23].

In healthy subjects, PEGV, a selective competitive GHR antagonist [33], decreases plasma IGF-I levels and increases blood GH concentrations [34]. Despite in vitro and in vivo studies have demonstrated a direct action of pegvisomant on different organs and tissues [35] and a possibile direct role in chemoresistance [36,37], data concerning direct effects of PEGV on GH secretion by pituitary adenoma are conflicting. Some studies have observed an impairment of GH autofeedback in somatotrophs [38,39], whereas other investigators have demonstrated that PEGV does not effect pituitary somatotrophs directly and it does not cross the human blood–brain barrier [40,41], thus favoring GH-secretion indirectly via IGF-I lowering.

In our study, the PEGV dose probably has to be progressively increased over time to maintain IGF-I levels within target ranges, particularly in the documented presence of residual GH-secreting tumor tissue. An “escape” phenomenon of this type has been reported by several groups [32,42,43]. Although still poorly defined, it has been linked to diverse factors, including distracted physicians, noncompliant patients, and intrinsic features of the adenoma itself [44]. In our opinion, it may also stem from the increasing GH hypersecretion documented during PEGV therapy [8,19]. In patients who are SSA-resistant and therefore have persistently high levels of GH and IGF-I produced by an aggressive type of adenoma, it is conceivable that the dose of PEGV (regardless of whether it is given alone or with an SSA) will have to be periodically increased over time to control rising GH production. This hypothesis naturally needs to be confirmed with additional studies in larger populations, but physicians should be aware that ongoing monitoring of treatment responses is essential, even after IGF-I normalization has been achieved.

## Conclusions

We found for the first time that, in SSA-refractory GH-pituitary tumours, combination therapy (PEGV?+?SSA) was more likely to be prescribed for patients with clinical/biochemical/imaging evidence of relatively severe/aggressive disease along with a more substantial (albeit incomplete) IGF-I response to SSA monotherapy (PEGV alone). Both regimens were well tolerated, and at the end of follow-up, there was no significant difference between the daily PEGV doses in the two groups. However, our data show that, in SSA-resistant acromegaly, the final PEGV dose increases with treatment duration and therefore, for all patients, special attention is required, as there is a need for continuous PEGV dose-adjustement during long-term follow-up.

## Competing interests

The authors declare that they have no competing interests.

## Authors’ contributions

AB and LDM had the idea for this research, took responsibility for the design of this work and wrote the manuscript. AB and FV performed all statistical analyses. FV, RI, MP, RB, MP, AG, LT, SC have made substantial contributions in acquisition of data, laboratory analyses and interpretation of data for each involved center. MA, PG, AF, VT, AP have been involved in revising critically the manuscript and have given final approval of the version to be published.
